# Draft genome sequence and annotation of *Priestia aryabhattai* MS3, a salt-tolerant plant growth-promoting rhizobacteria

**DOI:** 10.1128/mra.00026-25

**Published:** 2025-04-15

**Authors:** Dipta Chandra Pal, Shakila Nargis Khan, Muhammad Manjurul Karim

**Affiliations:** 1 Department of Microbiology, University of Dhaka 95324, Dhaka, Bangladesh; The University of Arizona, Tucson, Arizona, USA

**Keywords:** *Priestia aryabhattai*, PGPR, salt tolerance

## Abstract

*Priestia aryabhattai* strain MS3, a salt-tolerant, plant growth-promoting rhizobacterium, was isolated from saline soil in Kalapara, Patuakhali, Bangladesh. This study presents the whole-genome sequencing of strain MS3, revealing a 5.296 Mb genome comprising 5,369 predicted protein-coding sequences and 46 RNA genes.

## ANNOUNCEMENT

Plant growth-promoting rhizobacteria (PGPRs) play a pivotal role in sustainable agriculture by enhancing plant growth and resilience to environmental stresses (1). The genus *Priestia*, belonging to the family Bacillaceae, encompasses metabolically versatile and ecologically significant species. Among them, *Priestia aryabhattai*—a gram-positive rod formerly classified as *Bacillus aryabhattai* (MG209571.1) (2)—has gained prominence as a model organism for agricultural and biotechnological applications due to its adaptability and plant growth-promoting attributes.

*Priestia aryabhattai* strain MS3 exhibits key PGPR traits, including nitrogen fixation, phosphate solubilization, and phytohormone production (3). Field studies confirm its role in mitigating salt stress in rice by enhancing antioxidant defense, ion homeostasis, and photosynthesis (4). Its siderophore production under iron-limiting saline conditions highlights its adaptability (5). This study presents its draft genome, shedding light on its metabolic capabilities and ecological significance for sustainable agriculture.

*P. aryabhattai* strain MS3 was isolated from saline soil (electrical conductivity: 8.48 mS/cm, chloride: 0.38%, sodium: 15 mg/kg, and potassium: 1.02%) of Kalapara, Patuakhali, Bangladesh. Samples were collected from a depth of 50 to 100 mm, where microbial activity is most prominent and considered optimal for cropping (3, 6). A 1% (wt/vol) suspension of soil samples prepared in phosphate-buffered saline (pH 7.4) was added to selective Jensen broth medium (pH 7.2 ± 0.2 at 25°C) and incubated at 30°C at 120 rpm for 72 hours under aerobic conditions. After incubation, 100 µL of the sample was spread-plated onto Jensen agar and incubated at 30°C for 72–96 hours until colonies were detected. Genomic DNA was extracted from a pure culture grown on a tryptic soy agar plate using a QIAamp DNA Mini Kit (Qiagen, Germany). The purity and concentration of the extracted DNA were assessed with a Berthold Colibri Model LB 915 microvolume spectrophotometer (Berthold Technologies GmbH & Co. KG, Germany). Next-generation sequencing library preparation was performed using the Illumina DNA Prep kit, followed by adapter ligation using Illumina Nextera DNA CD Indexes, according to the manufacturer’s protocol, without any additional modifications (Illumina, San Diego, CA, USA). Sequencing was carried out on the Illumina NextSeq 550 platform, generating paired-end reads of 151 bp in length.

Whole-genome sequencing analysis was conducted using the KBase platform (7). Raw data quality was assessed with FastQC v.0.12.1 (8), and adapter regions and low-quality reads were filtered using Trimmomatic v.0.39 (9). A total of 1,370,833 read pairs were retained after filtering and trimming. Genome assembly was performed using SPAdes v.3.15.5 (10), and the quality of the assembly was evaluated with QUAST v.5.3.0 (11). Genome completeness was assessed with CheckM v.1.0.18 (12). Taxonomic assessment was conducted using GTDB-Tk v.2.4.0 (13), and secondary metabolite analysis was performed with antiSMASH v.6.1.1 (14). The annotation for prediction of gene functions was performed using National Center for Biotechnology Information Prokaryotic Genome Annotation Pipeline v.6.9 (15). All software tools were run with default parameters unless otherwise stated.

The draft genome of *P. aryabhattai* strain MS3 exhibited 55× coverage and 99.43% completeness, consisting of 42 contigs. GTDB-Tk confirmed a FastANI taxonomy score of 98.74% with *P. aryabhattai*. The annotated genome length, GC (guanine-cytosine) content, and N50 values were 5,296,295 bp, 37.77%, and 644,034, respectively(Table 1). Analysis of secondary metabolites revealed the presence of non-ribosomal peptide synthetase, RRE-containing clusters, siderophore, terpene, and Type III PKS.

**TABLE 1:** Whole-genome sequencing characteristics of *Priestia aryabhattai* strain MS3

Parameter^*a*^	*P. aryabhattai* strain MS3
Genome size (contigs >500 bp)	5,296,295
G + C contents (%) (bp)	37.77
Number of contigs	42
Total read pairs	1,370,833
Total base pair sequenced	404,909,146
N50 (bp)	644,034
N90 (bp)	115,847
L50	4
L90	11
FastANI taxonomy	98.74
Number of genes (total)	5,477
Number of CDSs (total)	5,431
Number of genes (coding)	5,369
Number of CDSs (with protein)	5,369
Number of genes (RNA)	46
Number of rRNAs	2, 1, and 2 (5S, 16S, and 23S)
Number of complete rRNAs	2 and 1 (5S and 16S)
Number of partial rRNAs	2 (23S)
Number of tRNAs	33
Number of ncRNAs	8
Number of pseudogenes (total)	62

^*a*^ C, cytosine; CDS, coding sequence; G, guanine.

## Data Availability

This Whole Genome Shotgun project has been deposited at DDBJ/ENA/GenBank under accession number JBKIWT000000000. The version described in this paper is version JBKIWT000000000.1. The raw sequencing data from this study have been deposited in the National Center for Biotechnology Information Sequence Read Archive under accession number SRR31905626 (BioProject accession number PRJNA1207046 and BioSample accession number SAMN46113985).
